# Altered Circadian Food Anticipatory Activity Rhythms in PACAP Receptor 1 (PAC1) Deficient Mice

**DOI:** 10.1371/journal.pone.0146981

**Published:** 2016-01-12

**Authors:** Jens Hannibal, Birgitte Georg, Jan Fahrenkrug

**Affiliations:** Department of Clinical Biochemistry, Faculty of Health Sciences, Bispebjerg Hospital, University of Copenhagen, Copenhagen, Denmark; Kent State University, UNITED STATES

## Abstract

Light signals from intrinsically photosensitive retinal ganglion cells (ipRGCs) entrain the circadian clock and regulate negative masking. Two neurotransmitters, glutamate and Pituitary Adenylate Cyclase Activating Polypeptide (PACAP), found in the ipRGCs transmit light signals to the brain via glutamate receptors and the specific PACAP type 1 (PAC1) receptor. Light entrainment occurs during the twilight zones and has little effect on clock phase during daytime. When nocturnal animals have access to food only for a few hours during the resting phase at daytime, they adapt behavior to the restricted feeding (RF) paradigm and show food anticipatory activity (FAA). A recent study in mice and rats demonstrating that light regulates FAA prompted us to investigate the role of PACAP/PAC1 signaling in the light mediated regulation of FAA. PAC1 receptor knock out (PAC1-/-) and wild type (PAC1+/+) mice placed in running wheels were examined in a full photoperiod (FPP) of 12:12 h light/dark (LD) and a skeleton photoperiod (SPP) 1:11:1:11 h L:DD:L:DD at 300 and 10 lux light intensity. Both PAC1-/- mice and PAC1+/+ littermates entrained to FPP and SPP at both light intensities. However, when placed in RF with access to food for 4–5 h during the subjective day, a significant change in behavior was observed in PAC1-/- mice compared to PAC1+/+ mice. While PAC1-/- mice showed similar FAA as PAC1+/+ animals in FPP at 300 lux, PAC1-/- mice demonstrated an advanced onset of FAA with a nearly 3-fold increase in amplitude compared to PAC1+/+ mice when placed in SPP at 300 lux. The same pattern of FAA was observed at 10 lux during both FPP and SPP. The present study indicates a role of PACAP/PAC1 signaling during light regulated FAA. Most likely, PACAP found in ipRGCs mediating non-image forming light information to the brain is involved.

## Introduction

The daily changes in behavior and physiology in mammals are driven by a biological clock located in the hypothalamic suprachiasmatic nucleus (SCN). A complex molecular machinery within neurons of the SCN generates a synchronized rhythm of approximately 24 h.Output signals from the SCN initiate and set the temporal niche of the sleep-wake cycle, feeding behavior, hormone secretion, temperature and heart rate regulation [1]. Importantly, central information from the SCN is able to synchronize clock driven rhythmicity within different organs and tissues to ensure optimal conditions for survival and reproduction [2]. The SCN rhythm is daily adjusted (entrained) to the astronomical day by light which is the most important “zeitgeber” for entrainment [3]. In mammals, light information to the SCN is processed by melanopsin containing intrinsically photosensitive retinal ganglion cells (ipRGCs) which also receives input from rods and cones [4]. The ipRGCs project as the retinohypothalamic tract (RHT) to the SCN and other brain areas [5]. Two neurotransmitters of the RHTglutamate and PACAP transmit light information via subtypes of glutamate receptors and the PACAP specific receptor (PAC1) on the SCN neurons [6].

In animals, light entrainment occurs during the twilight zones. At early night, light is able to slow down the speed of the clock causing phase delay while light in the late night/early morning speeds up the clock causing phase advances [7, 8]. Light has little effect on clock phase during the daytime, which is considered as a “dead zone” regarding light responsiveness [3]. There is evidence that light responsiveness during daytime depends on the metabolic status of the animal [9]. In a hypocaloric state caused by lack of food (restricted feeding, RF), the sensitivity of the SCN clock is altered and light has an effect on the clock phasing during the day [10]. Furthermore, lack of food can change behavior and physiology in a clock-controlled process independent of the SCN, and the existence of a food entrainable oscillator (FEO) has been suggested [11, 12]. The localization of the FEO is unknown but may consist of a network of neurons rather than a single group of neurons as found for the light entrainable oscillator (LEO) in the SCN [11].

In nocturnal animals, restricted daily feeding limited to a few hours during the resting/sleep phase of the day induces circadian food anticipatory activity (FAA), which is initiated in the hours before the meal is presented [13]. Under normal conditions, activity in nocturnal animals is suppressed during the light phase, a process known as negative or photic masking [14, 15]. In a recent study, Patton et al. (2013) investigated the role of light on FAA in rats and mice by comparing animals kept in a skeleton photoperiod (SPP) and a full photoperiod (FPP) and found that light had strong masking effect on FAA [16].

We have previously shown that PACAP/PAC1 signaling is involved in light entrainment and negative masking at night [17]. This prompted us to investigate the role of PACAP/PAC1 signaling on FAA by using mice lacking the PACAP specific PAC1 receptor. By comparing FPP and SPP at two light intensities (300 and 10 lux), we found that PACAP/PAC1 signaling is an important regulator of FAA.

## Material and Methods

### Animals

PAC1 receptor knockout (PAC1-/-) mice [17] were bred in our colony and used in the present experiments. Wild type (PAC1+/+) and PAC1-/- mice on a 129 background were bred from heterozygote animals and genotyped as described previously [18]. Mice were 10–12 weeks old when included in the experiments. All animals were maintained in a 12:12 h light/dark (LD) cycle housed in individual cages with water and food *ad libitum* (Altromin 1324; Altromin Spezialfutter, Germany) unless otherwise stated. All animals were treated according to the Ethical principles of Laboratory Animal Care (Law on Animal Experiments in Denmark, publication 1306, November 23, 2007). The study was approved by The Scientific Ethical Committee; Dyreforsoegstilsynet, Ministry of Justice, Denmark, who license the study by number: 2008/561-1445 to Jan Fahrenkrug, Head of the Department of Clinical Biochemistry, Bispebjerg Hospital. All animals were sacrificed by decapitation at the end of the study.

#### Measurement of running wheel activity rhythms

A total of 16 mice (8 PAC1+/+ and 8 PAC1-/- animals, four of each sex in each group) with food and water *ad libitum* were entrained to a FPP of 12:12 h light/dark (LD) cycle in individual cages equipped with a running wheel in ventilated, light-tight chambers with controlled white light. Wheel running activity was monitored by an on-line PC connected via a magnetic switch to the MiniMitter Running Wheel activity system (consisting of QA-4 activity input modules, DP-24 dataports and Vital View data acquisition system, MiniMitter Company, Inc. Sunriver, OR, USA vers. 4.1) [19]. Wheel revolutions were collected continuously in 6 minute bins. Animals were entrained to a 12:12 h LD cycle (lights on at 7:00 a.m. designated Zeitgeber time (ZT) = 0, off at 7.00 p.m. = ZT12) for at least 14 days before start of experiments.

#### Light source and light intensity measurements

White lightning was delivered from fluorescent tubes placed on top of each cage. The light intensity was adjusted from 10 to 900 lux via a resistance. Light intensity was measured using an Advantest Optical Power meter TQ8210 (MetricTest, Hayward, CA), and measurements were determined at settings of 514 nm; 300 lux (115.0 μW/cm^2^) and 10 lux (4.3 μW/cm^2^), respectively.

### Experimental design

#### Light and feeding schedules

**Fig 1:**
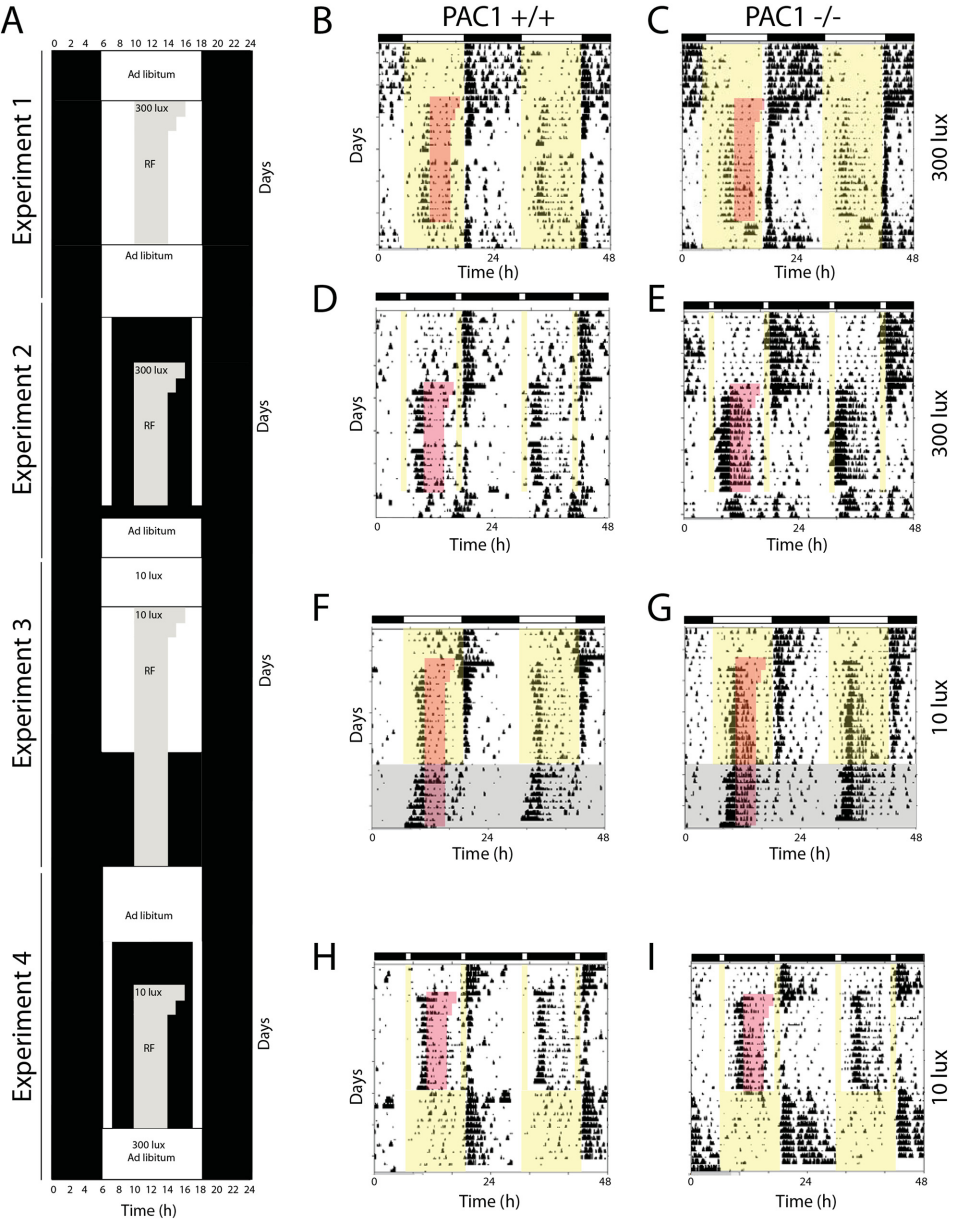
Food anticipatory activity (FAA) during restricted feeding (RF) and different light conditions. A. Restricted feeding protocols with food deprivation during the daytime starting at ZT 4 in PAC1 +/+ and PAC1-/- mice were fed ad libitum and then the availability of food were gradually reduced (RF) from 6 h/day to 4 h/day (ZT4—ZT8). The RF time during each experiment (1–4) is indicated by gray shading, light in white and dark in black. Experiment 1 was performed with light in a full photoperiod (FPP) of 12:12 h light/darkness (L/D) at 300 lux. Experiment 2 was performed in a skeleton photoperiod (SPP) of 1:10:1:12 h L/D/L/D at 300 lux. Experiment 3 was performed with light in a full photoperiod (FPP) of 12:12 h L/D at 10 lux followed by a period of DD for 10 days. Experiment 4 was performed in a skeleton photoperiod (SPP) of 1:10:1:12 h L/D/L/D at 10 lux. Representative actograms from PAC1+/+ and PAC1-/- mice in Experiment 1 are shown in B-C, Experiment 2 in D-E, Experiment 3 in F-G, and Experiment 4 in H-I. In B-I RF is indicated by red shading, light periods by yellow shading and constant darkness in Experiment 3 by gray shading. The light-dark cycle is indicated by the white and black bars on top of each actogram, respectively.

The light and feeding schedule for Experiment 1–4 is shown in Fig 1. PAC1+/+ and PAC1-/- mice were assigned to FPP or SPP with initial feeding and water *ad libitum* followed by a period of RF. FPP consisted of 12:12 h LD. SPP consisted of 1 h light (ZT0—ZT1 i.e. 6–7 am), 10 h darkness, 1 h light (ZT11—ZT12 i.e. 6–7 pm). Experiment 1–4 were initiated after animals were stably entrained at 300 lux for at least 14 days. After each experiment, animals received food and water *ad libitum* and stable entrainment at the given light conditions was achieved within 14 days. All mice were exposed for RF for 16–19 days. A schematic illustration of the experimental conditions is shown in Fig 1A. **Experiment 1**: FPP at 300 lux; RF at 300 lux followed by reentrainment in FPP at 300 lux, feeding *ad libitum*. **Experiments 2**: SPP at 300 lux; RF in SPP at 300 lux followed by reentrainment in FPP at 300 lux, feeding *ad libitum*. **Experiment 3**: FPP at 10 lux; RF at 10 lux. In this experiment, animals were kept for another 10 days at RF but light was turned off to investigate the degree of food entrainment during constant darkness. **Experiment 4**: SPP at 10 lux; RF in SPP at 10 lux, feeding *ad libitum*.

Restricted feeding schedules were initiated by a gradual adaptation to the restricted feeding paradigm by presenting the food from ZT4 to ZT10 for the first two days followed by 2–3 days with food present from ZT2 to ZT9, finally by a 4 h restricted feeding period from ZT4 to ZT8 (Fig 1A). Such a paradigm has previously been show useful to eliminate death during RF [20]. During RF all animals were weighed every second day. If mice showed signs of weakness and/or a weight loss of more than 5–10% from the previous weight, the food was placed in the bottom of the cages instead of in the food locker. Stable FAA was found typically after 3–6 days. For assessment of daily activity pattern of wheel-running activity, the cumulative wheel revolutions performed during the last 5 days in each condition were recorded for each animal. The average of these 5 days (for each animal) was used to analyze activity pattern for the genotypes during the various paradigms [13]. The onset of FAA was calculated from the same days using the onset module in ClockLab (ActiMetric Software, Coulbourn Instruments, Wilmette, IL, USA). The time points from the 5 days were averaged (for each animal), and time from onset to meal time in minutes were used to determine the differences in onset between the two genotypes. All data obtained from *the Minimitter Running Wheel activity system* were analyzed in ClockLab (ActiMetric Software, Coulbourn Instruments, Wilmette, IL, USA) running under Matlab (v. R2012a, 64-bit for Windows7, MathWorks, Natick, MA, USA) environment. Data generated in ClockLab were saved in a spreadsheet and plotted as an average activity cycle using GraphPad Prism 5.0 (GraphPad Software, Inc. La Jolla, CA, USA). Statistics were performed using GraphPad Prism. For comparison of two independent groups, unpaired t-test was used. *P* < 0.05 was considered statistically significant. Bonferroni correction was used on FAA activity to evaluate family-wise errors.

Figure plates were mounted in Adobe Illustrator CS5 (Adobe System Incorporated, San Jose, CA, USA).

## Results

### Experiment 1. Full photoperiod and restricted feeding at 300 lux

**Fig 2:**
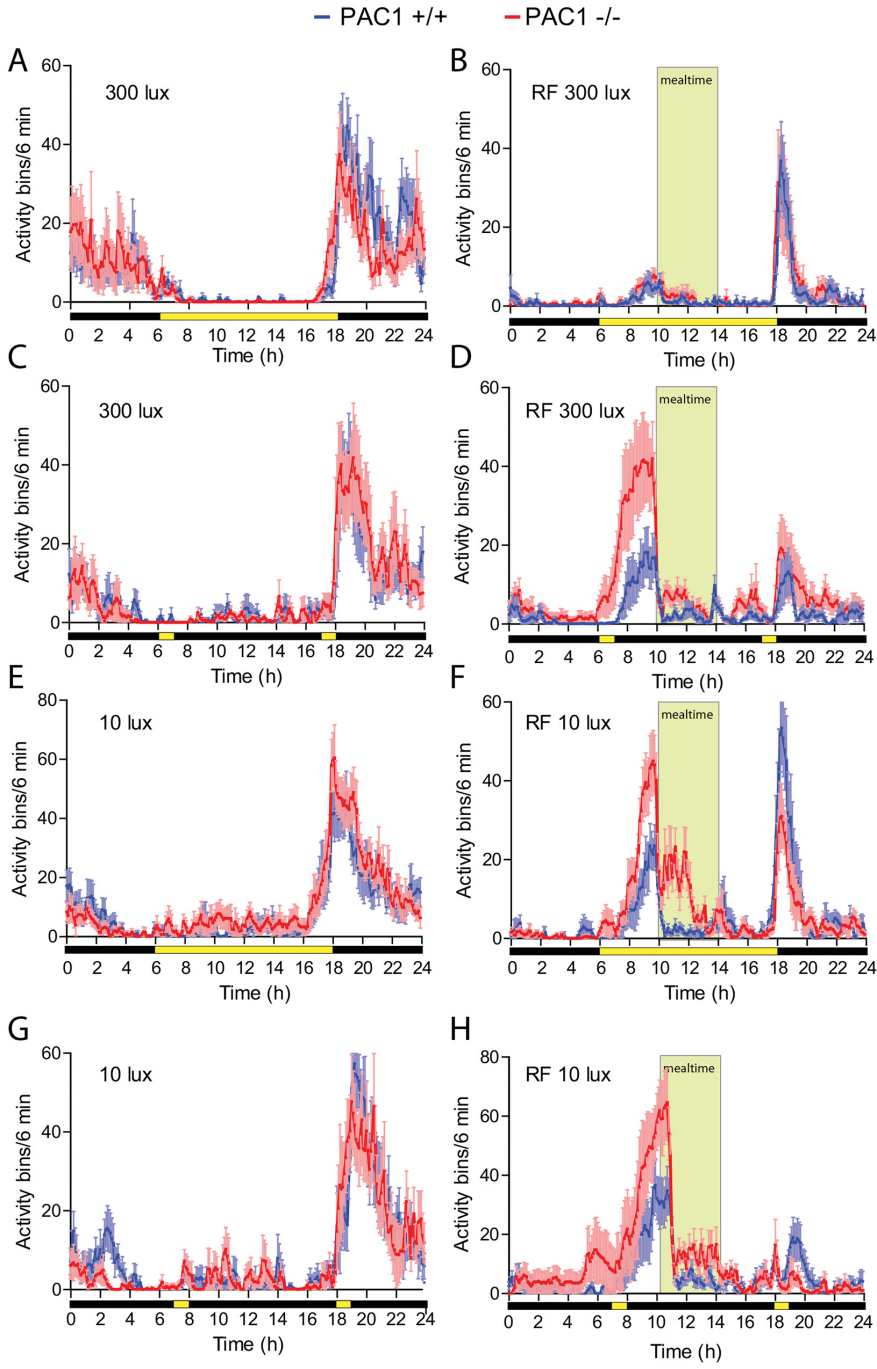
Food anticipatory activity (FAA) in PAC1+/+ and PAC1-/- receptor deficient mice under a full photoperiod (FPP) and skeleton photoperiod (SPP) at light intensities of either 300 lux or 10 lux. The light/dark period is indicated below the X-axis in yellow/black. The same 8 animals of each genotype were used in all experiments and data are average of 5 d of activity (see also material and methods). A. Group mean (± SEM) waveform of wheel running activity of PAC1+/+ (blue line) and PAC1-/- mice (red line) during FPP at 300 lux. B. Group mean (± SEM) waveform of wheel running activity of the same animals as in A showing FAA before mealtime (indicated by light green). C. Group mean (± SEM) waveform of wheel running activity of PAC1+/+ (blue line) and PAC1 -/- mice (red line) during SPP at 300 lux. D. Group mean (± SEM) waveform of wheel running activity of the same animals as in C showing FAA before mealtime (indicated by light green). E and F correspond to A and B but at light intensity of 10 lux. G and H correspond to C and D but at light intensity of 10 lux.

**Fig 3:**
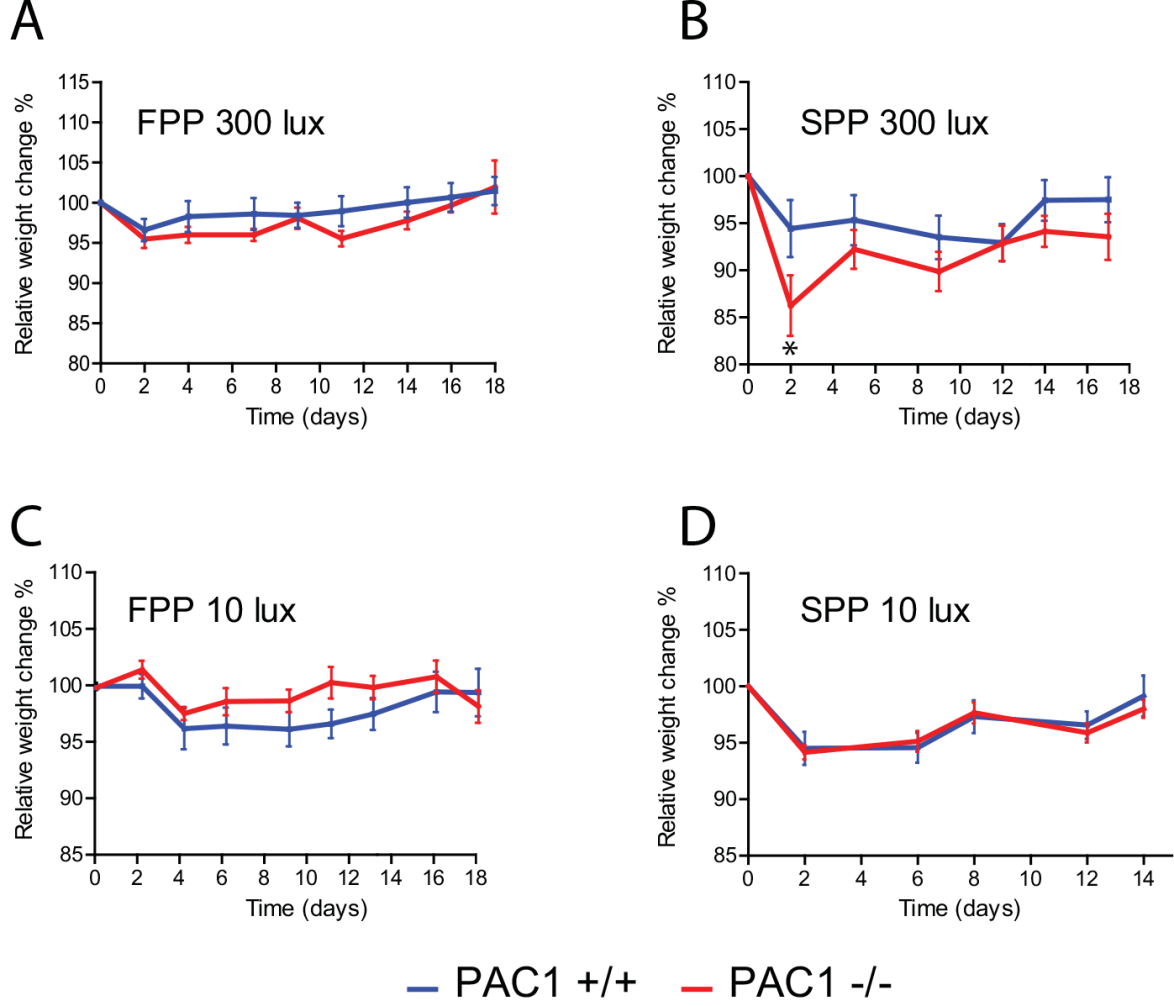
A-D. Weight profiles (mean ± SEM) of during RF regime of PAC1+/+ (PAC1+/+) (blue line) and PAC1 (PAC1-/-) receptor deficient mice (red line) shown in Fig 2. Start weight was set to 100% and the mean of the relative weight change every second day during RF for each animal of the two groups was plotted.

Access to *ad libitum* food resulted in PAC1+/+ and PAC1-/- mice entrainment to the LD cycle and no significant differences were found in neither phase nor activity between the two genotypes (Fig 1B and 1C, Fig 2A, Table 1). During RF at FPP both genotypes showed significantly reduced activity compared to activity during *ad libitum* feeding (PAC1+/+: 2091 ± 388 vs. 676 ± 194; *p* = 0.008 and PAC1-/-: 1907 ± 620 vs. 582 ± 124; *p* = 0.05, Table 1). All animals demonstrated FAA during RF with no difference between the two genotypes (Fig 1B and 1C, Fig 2B, Table 1). FAA was seen from approximately 2 h before mealtime and lasted 1–2 h into the meal time period (Fig 1B and 1C, Fig 2B). The main activity during FPP at 300 lux was at subjective night (Fig 1B and 1C, Fig 2A and 2B, Table 1). During RF, weight loss seemed more pronounced in PAC1-/- mice but did not differ significantly from PAC1+/+ mice and was overall less than 5% of the initial weight (Fig 3A). In both genotypes initial body weight was reached at the end of the RF regime (Fig 3A).

**Table 1:** Activity at full (FPP) and skeleton (SPP) photoperiods in PAC1+/+ and PAC1-/- mice.

300 lux FPP	PAC1 +/+	PAC1 -/-	*p*-value
Total activity FPP	2091±388	1907±620	
Total activity RF FPP	676±194	582±124	
Night activity (ZT12-24) RF FPP	444±135	419±101	
FAA (ZT06-10) RF FPP	116±30	78±26	
**300 lux SPP**			
Total activity SPP	1591±391	1730±555	
Total activity RF SPP	762±182	2272±714	*p* = 0.049 *
Night activity (ZT12-24) RF SPP	268±87	703±327	
FAA (ZT06-10) RF SPP	306±119	1159±342	*p* = 0.027 *
**10 lux FPP**			
Total activity FPP	2156±371	2829±435	
Total activity RF FPP	1303±166	1942±216	*p* = 0.037 *
Night activity (ZT12-24) RF FPP	711±145	500±124	
FAA (ZT06-10) RF FPP	327±106	794±114	*p* = 0.011 *
**10 lux SPP**			
Total activity SPP	2057±308	2139±517	
Total activity RF SPP	1542±373	2584±724	
Night activity (ZT12-24) RF SPP	397±138	516±241	
FAA (ZT06-10) RF SPP	759±265	1438±442	
**DD after 10 lux FPP**			
Total activity	2073±397	2296±688	
Total activity RF FPP	1113±120	3013±310	*p* = 0.0001***
“Night activity” (ZT12-24) RF FPP	178±75	568±175	
FAA (ZT06-10) RF FPP	619±149	1564±179	*p* = 0.0016**

### Experiment 2. Skeleton photoperiod and restricted feeding at 300 lux

Both PAC1+/+ and PAC1-/- mice exposed to *ad libitum* feeding entrained to SPP at 300 lux displaying an activity profile very similar to that observed at FPP (Fig 1D and 1E, Fig 2C vs. 2A). No significant change was observed in total activity comparing FPP and SPP of either genotypes on *ad libitum* feeding (Table 1). However, during RF PAC1-/- mice displayed a significant change in activity compared to PAC1+/+ mice. Both PAC1+/+ and PAC1-/- mice demonstrated FAA with both genotypes showing higher FAA during SPP in comparison to FPP (PAC1 +/+: 306 ± 119 vs. 116 ± 30; *p* = 0.172 and PAC1 -/-: 1159 ± 342 vs. 78 ± 26; *p*<0.0001, Table 1). Although, FAA in PAC1KO mice was significantly higher compared to PAC1+/+ mice (1159 ± 341 vs. 306 ± 119; *p* = 0.027)(Fig 1D and 1E, Fig 2D vs. 2B, Table 1). Furthermore, the onset of FAA was significantly advanced in PAC1 -/- mice (175 ± 9 min) compared to PAC1+/+ mice (97.5 ± 8.9 min; *p*<0.0001) (Fig 1D and 1E). Total activity during RF was also significantly higher in PAC1-/- mice compared to PAC1+/+ animals (2272 ± 714 vs. 762 ± 182; *p* = 0.049) (Table 1) and compared to FPP (2272 ± 714 vs. 582 ± 124; *p* = 0.027) due to the marked increase during FAA and higher night time activity. The activity during the mealtime period and until the evening light pulse did not differ between the genotypes. Weight loss during SPP was significantly larger in PAC1-/- mice compared to PAC1+/+ mice during the initial part of RF (Fig 3B). Hereafter a gradual weight gain was observed for both genotypes (Fig 3B). Due to the weight loss observed during the RF period, mice had food placed in the bottom of the cages. None of the genotypes gained weight to fully compensate for the weight loss during the RF period (Fig 3B).

### Experiment 3. Full photoperiod and restricted feeding at 10 lux

During *ad libitum* food access, both PAC1+/+ and PAC1-/- entrained to the LD cycle at 10 lux, and neither total activity (Table 1) nor activity onset was found to differ between the two genotypes (Fig 1F and 1G, Fig 2E). During FPP at RF at 10 lux both PAC1+/+ and PAC1-/- mice demonstrated FAA, and both genotypes showed increased FAA during FPP at 10 lux in comparison to FPP at 300 lux (PAC1 +/+: 327 ± 106 vs. 116 ± 30; *p* = 0.079 and PAC-/-: 794 ± 114 vs. 78 ± 26; *p*<0.0001, Figs 2F vs. 1B). The FAA activity of PAC1-/- mice was significantly higher than PAC1+/+ mice (Fig 1F and 1G, Fig 2F, 794 ± 114 vs. 327 ± 106; *p* = 0.011, Table 1). Onset of FAA was significantly advanced in PAC1-/- mice compared to PAC1+/+ mice (142 ± 10 min vs. 100 ± 5 min; *p* = 0.0025) (Fig 2F) and the activity continued during the mealtime (Fig 2F). In both genotypes, the total activity during RF at FPP at 10 lux was significantly increased compared to FPP at 300 lux (PAC1 +/+: 1303 ± 166 vs. 676 ± 194; *p* = 0.030 and PAC1-/-: 1942 ± 216 vs. 582 ± 124; *p*<0.0001, Table 1) being higher in PAC1-/- mice than in PAC1+/+ mice (1942 ± 216 vs. 1303 ± 166; *p* = 0.037, Table 1). Nighttime activity during RF did not differ between the two genotypes, no significant difference in weight loss was found and the initial weight loss was recovered during the RF period (Fig 3C).

After RF and FPP at 10 lux, PAC1+/+ and PAC1-/- mice were maintained on RF but transferred to constant darkness for another 10 days to evaluate whether the activity continued with similar pattern of FAA as seen during FPP at 10 lux (Fig 1F and 1G). In constant darkness, the SCN driven nocturnal activity gradually decreased in the first week (Fig 1F and 1G), but the FAA activity increased for both genotypes and reached the same activity level as found during RF at SPP at 10 lux (PAC1 +/+: 619 ± 149 and PAC1-/-: 1564 ± 179; *p*<0.0016, Table 1) suggesting that the animals primarily entrained to the feeding schedule during the last week of the DD period (Fig 1F and 1G).

### Experiment 4. Skeleton photoperiod and restricted feeding at 10 lux

Both PAC1+/+ and PAC1-/- mice entrained to SPP at 10 lux (Fig 1H–1I, Fig 2G) with activity profile similar to that found during FPP at 10 lux and at SPP at 300 lux (Fig 2C and 2E). When exposed to RF during SPP at 10 lux, FAA was increased in both genotypes similar to that seen at Experiment 2 and 3. The onset of PAC1-/- compared to PAC1+/+ animals seemed advanced although statistical significance was not reached (158 ± 20 min vs. 133 ± 15 min; *p* = 0.23). Similarly, FAA seemed larger in PAC1-/- compared to PAC1+/+ mice. However, due to large variation in FAA the difference was not significant (1438 ± 442 vs. 759 ± 265; *p* = 0.192, Table 1). During this regime, nocturnal activity was low and comparable to SPP at 300 lux (Fig 1H–1I, Fig 2D and 2H, Table 1). Weight loss reached 6–7% after 2 days during RF for both genotypes and both genotypes recovered this weight loss during the RF period (Fig 3D).

## Discussion

The present study demonstrates that light exposure during daytime can modulate FFA, which is an output of a putative FEO (16), and that PACAP via the PAC1 receptor plays a role in the light regulated FFA during the daytime. PACAP/PAC1 signaling seems to be most pronounced during a full photoperiod (FPP; parametric light sampling) at low light intensity and at skeleton photoperiods (SPP; non-parametric light sampling) at both high and low light intensity.

At daytime, nocturnal animals usually rest or sleep due to negative masking and sleep promoting signals from sleep areas in the brain. Sleep promoting areas in the ventral hypothalamus are influenced by the circadian clock and by light via the retinohypothalamic tract [21, 22]. However, RF during daytime induces FAA when the animal is asleep or at rest [9, 12]. The potential modulatory effects of light on FAA was recently addressed by Patton et al. (2013) who clearly demonstrated that light can modulate hypocaloric-induced FAA during the subjective day (16). In accordance, we found increased FAA in PAC1+/+ mice during SPP at 300 lux compared to FPP. Furthermore, we demonstrated that FAA activity was significantly increased and phase advanced in PAC1-/- compared to PAC1+/+ mice. This pattern was also found when the light intensity was reduced to 10 lux during FPP. This new information indicates that both light intensity as well as time of light stimulation i.e. FPP vs. SPP are important when monitoring FAA. Our results indicate that PACAP/PAC1 signaling is involved in light recording in the brain both -during parametric light conditions (FPP) at low light intensity and non-parametric light sampling (SPP) at both high and low light intensity. We are aware that a potential risk of carry over effects may occur using the same animals for all four experiments. However, similar experiments (unpublished) were performed on another group of PAC1 +/+ and -/- mice placed directly in SPP, and these mice showed similar changes in FAA corroborating that it is the experimental (light) regimes that drive the altered behavior.

PACAP, a neurotransmitter in the RHT, has been shown to play a role in light entrainment of the circadian clock and in negative masking [6, 17]. It is therefore likely that absence of PACAP/PAC1 signaling to retinal target areas in the brain directly or indirectly influences FAA. One possible target could be sleep promoting areas of the brain like the VLPO [23]. Light promotes sleep via the VLPO [21] and lack of PACAP/PAC1 signaling to the sleep activating neurons of the VLPO may change the light induced sleep drive and promote wakefulness. The intergeniculate leaflet of the lateral geniculate complex (IGL) or the SCN could also be the target area for an interaction between light/PACAP/PAC1 signaling and arousal signals involving orexin containing neurons innervating the IGL or the SCN [24–26].

The PAC1-/- mice used in this study are general KO animals. Since PACAP and its receptor are widely distributed in the central and peripheral nervous system the site(s) and action and mechanism(s) involved remain to be clarified. PACAP/PAC1 signaling has previously been shown to play a role in feeding regulation as an anorexigen neurotransmitter [27–29]. Both PAC1+/+ and PAC1 -/- mice demonstrate similar weight loss during RF indicating that this paradigm leads to a hypocaloric state during most of the experimental period. However, since weight loss in the two genotypes did not differ, PACAP/PAC1 signaling does not seem to influence weight balance [29]. PACAP located in TRH expressing neurons of the hypothalamic paraventricular nucleus [30] has been shown to be important for excitatory input to agouti-related peptide neurons of the arcuate nucleus driving food seeking behavior [31]. We found that food seeking behavior (FAA) was increased, not decreased proving less likely that this extra retinal PACAP/PAC1 signaling is involved in light induced regulation of FAA.

In support of a retinal PACAP driven mechanism are two previous studies in rats treated with neonatally administered monosodium glutamate (MSG). MSG treatment leads to severe retinal degeneration of the retinal ganglion cell layer and a marked reduction in PACAP projections from the eye in neonatal MSG treated rats [32]. Furthermore, neonatal MSG treated rats demonstrated altered FAA during light stimulation corroborating our findings in PAC1-/- mice [33]. This could support the notion that the altered FAA observed in PAC1 -/- mice is due to lack of PACAP/PAC1 signals from the eyes.

During *ad libitum* feeding light has little or no effect on circadian phase regulation during the subjective day [7]. This daytime insensitivity to light is a fundamental property of the LEO in the SCN [3]. However, light seems to play a more prominent role at RF than previously recognized [9, 12]. SCN sensitivity to light stimulation during daytime is altered by reduced food availability [10]. Altered light sensitivity due to a hypo-metabolic state has also been found to change clock sensitivity to light stimulation at night by mechanisms involving several clock genes [10, 34]. Thus, the altered FAA in PAC1-/-mice could be due to changed light sensitivity of the LEO and/or a result of masking by light mediated PACAP/PAC1 signaling.

Several years ago, we showed that PACAP had a direct effect on the SCN phase during the subjective day *in vitro* [35]. This finding was difficult to interpret since light has no effect on the circadian phase *in vivo* during daytime [7]. However, the brain slice model used was devoid of extrinsic innervation to the SCN and this might have altered the *in vitro* phase responsivity to PACAP similar to what has been seen for light responsivity during restricted feeding [10].

In natural life, nocturnal animals are photoentrained by light during the twilight zones which under experimental conditions can be simulated by SPP. The circadian timing system integrates information of day length by recording light during the daytime and there is evidence that both the SCN and the IGL are involved [3]. Both structures express the PAC1 receptor [23, 36] and are directly innervated by PACAP containing nerve fibers originating from melanopsin expressing ipRGCs [23]. Neurons in the IGL which project to the SCN via the geniculo-hypothalamic tract (GHT) [37] seem necessary for the animal to entrain to SPP [38]. It is possible that the altered FAA behavior found during SPP is caused by lack of PACAP/PAC1 signaling in these IGL neurons.

In conclusion, our study of FAA in PAC1-/- mice indicates a role of PACAP/PAC1 signaling during light regulated FAA. Most likely, PACAP found in ipRGCs mediating non-image forming light information to the brain, is involved.

## References

[1] Mohawk JA, Takahashi JS. Cell autonomy and synchrony of suprachiasmatic nucleus circadian oscillators. Trends Neurosci. 2011;34(7):10. 21665298 10.1016/j.tins.2011.05.003 PMC3775330

[2] Mohawk JA, Green CB, Takahashi JS. Central and peripheral circadian clocks in mammals. Annu Rev Neurosci. 2012;35:445–62. 10.1146/annurev-neuro-060909-153128 22483041 PMC3710582

[3] Golombek DA, Rosenstein RE. Physiology of circadian entrainment. Physiol Rev. 2010;90(3):1063–102. 10.1152/physrev.00009.2009 20664079

[4] Sand A, Schmidt TM, Kofuji P. Diverse types of ganglion cell photoreceptors in the mammalian retina. Prog Retin Eye Res. 2012. 10.1016/j.preteyeres.2012.03.003 PMC3361613 22480975

[5] Do MT, Yau KW. Intrinsically photosensitive retinal ganglion cells. Physiol Rev. 2010;90(4):1547–81. 10.1152/physrev.00013.2010 20959623 PMC4374737

[6] Hannibal J. Roles of PACAP-containing retinal ganglion cells in circadian timing. Int Rev Cytol. 2006;251:1–39. 16939776 10.1016/S0074-7696(06)51001-0

[7] Daan S, Pittendrigh CS. A functional analysis of the circadian pacemakers in nocturnal rodents. IV. Entrainment: Pacemaker and clock. J Comp Physiol. 1976;106:253–66.

[8] Daan S, Aschoff J. The entrainment of circadian systems In: Takahashi JS, Turek FW, Moore RY, editors. Circadian Clocks. Handbook of behavioral Neurobiology. 12: Kluwer Academic/Plenum Publisher; 2001 p. 7–43.

[9] Challet E. Interactions between light, mealtime and calorie restriction to control daily timing in mammals. J Comp Physiol B. 2010;180(5):631–44. 10.1007/s00360-010-0451-4 20174808

[10] Mendoza J, Graff C, Dardente H, Pevet P, Challet E. Feeding cues alter clock gene oscillations and photic responses in the suprachiasmatic nuclei of mice exposed to a light/dark cycle. J Neurosci. 2005;25(6):1514–22. 15703405 10.1523/JNEUROSCI.4397-04.2005 PMC6725981

[11] Davidson AJ. Lesion studies targeting food-anticipatory activity. Eur J Neurosci. 2009;30(9):1658–64. 10.1111/j.1460-9568.2009.06961.x 19863659

[12] Mistlberger RE. Neurobiology of food anticipatory circadian rhythms. Physiol Behav. 2011;104(4):535–45. 10.1016/j.physbeh.2011.04.015 21527266

[13] Mistlberger RE. Food-anticipatory circadian rhythms: concepts and methods. Eur J Neurosci. 2009;30(9):1718–29. 10.1111/j.1460-9568.2009.06965.x 19878279

[14] Redlin U. Neural basis and biological function of masking by light in mammals: suppression of melatonin and locomotor activity. Chronobiol Int. 2001;18(5):737–58. 11763983 10.1081/cbi-100107511

[15] Mrosovsky N. Masking: history, definitions, and measurement. Chronobiol Int. 1999;16(4):415–29. 10442236 10.3109/07420529908998717

[16] Patton DF, Parfyonov M, Gourmelen S, Opiol H, Pavlovski I, Marchant EG, et al Photic and pineal modulation of food anticipatory circadian activity rhythms in rodents. PLoSONE. 2013;8(12):e81588. 10.1371/journal.pone.0081588 PMC3852709 24324709

[17] Hannibal J, Brabet P, Fahrenkrug J. Mice lacking the PACAP type I receptor have impaired photic entrainment and negative masking. Am J Physiol Regul Integr Comp Physiol. 2008;295(6):R2050–R8. 10.1152/ajpregu.90563.2008 18922961

[18] Jamen F, Persson K, Bertrand G, Rodriguez-Henche N, Puech R, Bockaert J, et al PAC1 receptor-deficient mice display impaired insulinotropic response to glucose and reduced glucose tolerance. J Clin Invest. 2000;105(9):1307–15. 10792006 10.1172/JCI9387 PMC315446

[19] Hannibal J, Brabet P, Jamen F, Nielsen HS, Journot L, Fahrenkrug J. Dissociation between light induced phase shift of the circadian rhythm and clock gene expression in mice lacking the PACAP type 1 receptor (PAC1). J Neurosci,. 2001;21(13):4883–90. 11425915 10.1523/JNEUROSCI.21-13-04883.2001 PMC6762353

[20] Pendergast JS, Nakamura W, Friday RC, Hatanaka F, Takumi T, Yamazaki S. Robust food anticipatory activity in BMAL1-deficient mice. PLoSONE. 2009;4(3):e4860. 10.1371/journal.pone.0004860 PMC2654093 19300505

[21] Saper CB, Scammell TE, Lu J. Hypothalamic regulation of sleep and circadian rhythms. Nature. 2005;437(7063):1257–63. 16251950 10.1038/nature04284

[22] Morin LP. Nocturnal light and nocturnal rodents: similar regulation of disparate functions? J Biol Rhythms. 2013;28(2):95–106. 10.1177/0748730413481921 23606609

[23] Hannibal J, Fahrenkrug J. Target areas innervated by PACAP immunoreactive retinal ganglion cells. Cell Tissue Res. 2004;316:99–113. 14991397 10.1007/s00441-004-0858-x

[24] Marston OJ, Williams RH, Canal MM, Samuels RE, Upton N, Piggins HD. Circadian and dark-pulse activation of orexin/hypocretin neurons. Molecular brain. 2008;1:19 10.1186/1756-6606-1-19 19055781 PMC2632999

[25] Cutler DJ, Morris R, Sheridhar V, Wattam TA, Holmes S, Patel S, et al Differential distribution of orexin-A and orexin-B immunoreactivity in the rat brain and spinal cord. Peptides. 1999;20(12):1455–70. 10698122 10.1016/s0196-9781(99)00157-6

[26] Peyron C, Tighe DK, van den Pol AN, de Lecea L, Heller HC, Sutcliffe JG, et al Neurons containing hypocretin (orexin) project to multiple neuronal systems. J Neurosci. 1998;18(23):9996–10015. 9822755 10.1523/JNEUROSCI.18-23-09996.1998 PMC6793310

[27] Resch JM, Maunze B, Phillips KA, Choi S. Inhibition of food intake by PACAP in the hypothalamic ventromedial nuclei is mediated by NMDA receptors. Physiol Behav. 2014;133:230–5. 10.1016/j.physbeh.2014.05.029 24878316 PMC4126770

[28] Iemolo A, Ferragud A, Cottone P, Sabino V. Pituitary Adenylate Cyclase-Activating Peptide in the Central Amygdala Causes Anorexia and Body Weight Loss via the Melanocortin and the TrkB Systems. Neuropsychopharmacol. 2015; 40:1846–55. 10.1038/npp.2015.34 PMC4839508 25649277

[29] Matsuda K, Azuma M, Maruyama K, Shioda S. Neuroendocrine control of feeding behavior and psychomotor activity by pituitary adenylate cyclase-activating polypeptide (PACAP) in vertebrates. Obes Res Clin Pract. 2013;7(1):e1–e7. 10.1016/j.orcp.2012.10.002 24331677

[30] Legradi G, Hannibal J, Lechan RM. Association between pituitary adenylate cyclase activation polypeptide and thyrotropin-releasing hormone in the rat hypothalamus. J Chem Neuroanat. 1997;13:265–79. 9412908 10.1016/s0891-0618(97)10002-3

[31] Krashes MJ, Shah BP, Madara JC, Olson DP, Strochlic DE, Garfield AS, et al An excitatory paraventricular nucleus to AgRP neuron circuit that drives hunger. Nature. 2014;507(7491):238–42. 10.1038/nature12956 24487620 PMC3955843

[32] Hannibal J, Vrang N, Card JP, Fahrenkrug J. Light dependent induction of c-Fos during subjective day and night in PACAP containing retinal ganglion cells of the retino-hypothalmic tract. J Biol Rhythms. 2001;16:457–70. 11669419 10.1177/074873001129002132

[33] Mistlberger RE, Antle MC. Neonatal monosodium glutamate alters circadian organization of feeding, food anticipatory activity and photic masking in the rat. Brain Res. 1999;842(1):73–83. 10526097 10.1016/s0006-8993(99)01836-3

[34] Mendoza J, Pevet P, Challet E. Circadian and photic regulation of clock and clock-controlled proteins in the suprachiasmatic nuclei of calorie-restricted mice. Eur J Neurosci. 2007;25(12):3691–701. 17610588 10.1111/j.1460-9568.2007.05626.x

[35] Hannibal J, Ding JM, Chen D, Fahrenkrug J, Larsen PJ, Gillette MU, et al Pituitary adenylate cyclase activating peptide (PACAP) in the retinohypothalamic tract. A daytime regulator of the biological clock. J Neurosci. 1997;17:2637–44. 9065523 10.1523/JNEUROSCI.17-07-02637.1997 PMC6573509

[36] Engelund A, Fahrenkrug J, Harrison A, Luuk H, Hannibal J. Altered pupillary light reflex in PACAP receptor 1-deficient mice. Brain Res. 2012;1453:17–25. 10.1016/j.brainres.2012.03.005 22459045

[37] Hannibal J, Fahrenkrug J. Neuronal input pathways to the brain's biological clock and their functional significance. Adv Anat Embryol Cell Biol. 2006;182:1–71. 16566431

[38] Edelstein K, Amir S. The role of the intergeniculate leaflet in entrainment of circadian rhythms to a skeleton photoperiod. J Neurosci. 1999;19(1):372–80. 9870966 10.1523/JNEUROSCI.19-01-00372.1999 PMC6782384

